# Mapping end-of-life care in India: a scoping review to identify gaps in policy, practice, and psychosocial support

**DOI:** 10.1186/s12904-025-01825-z

**Published:** 2025-07-07

**Authors:** Babita P. A. Varkey, Arun Ghoshal, Naveen Salins, Catriona R. Mayland

**Affiliations:** 1 Karunashraya - Bangalore Hospice Trust, Old Airport Varthur Main Road, Kundalahalli Gate, Marathahalli, Bangalore, 560037 Karnataka India; 2 Department of Palliative Medicine and Supportive Care, Kasturba Medical College Manipal, Manipal Academy of Higher Education, Manipal, 576104 Karnataka India; 3 School of Medicine and Population Health, University of Sheffield, Royal Hallamshire Hospital, Glossop Road, Sheffield, S10 2RX UK

**Keywords:** Terminal care, Hospice care, Palliative care, Caregivers, India

## Abstract

**Background:**

Little about access to palliative and end-of-life care in India is known.

**Aim:**

To map various facets of end-of-life care in India, from perceptions of stakeholders to capacity and quality of care, training, and education, and to identify the current gaps in end-of-life care delivery.

**Design:**

A scoping literature review was conducted, with the protocol registered on the Open Science Framework, on November 29, 2023 (https://osf.io/twc9j).

**Data sources:**

Between January 1, 1990, and May 31, 2024, an electronic literature search was conducted using the MEDLINE, SCOPUS, CINAHL, EMBASE, and PSYCHINFO databases, as well as citations and grey literature.

**Results:**

The availability and accessibility of end-of-life care are limited to a few geographical regions, primarily urban areas. While some states have community-based programs, most end-of-life care practices are concentrated in hospitals, especially intensive care units. Patients frequently lack access to essential medications, such as morphine, as well as appropriately trained medical professionals and adequate infrastructure. Financial difficulties, limited knowledge, social stigma toward the terminally ill and dying, and the psychological and physical burdens of care add to the challenges faced by stakeholders.

**Conclusion:**

The availability and accessibility of end-of-life care in India are fragmented. A comprehensive strategy that includes policy and legislative reforms, education, and expanded palliative services is crucial for improving the quality of end-of-life care across the country.

**Supplementary Information:**

The online version contains supplementary material available at 10.1186/s12904-025-01825-z.

## Key statements

What is already known about the topic?

• Access to palliative and end-of-life care is limited in India; it is fragmented and concentrated in a few geographical areas, and predominant support is provided for those suffering from cancer.

• Futile interventions, especially in tertiary care centers, are often practiced for patients in end-of-life care, or they are discharged home to die due to legal barriers and fear of prosecution under the law.

• Poor financial and psycho-social support is a major component of the palliative and end-of-life scenario in India.

What does this paper add?

• Incorporating perspectives from patients, caregivers, and healthcare professionals, several key gaps have been identified in end-of-life care. These include a lack of adequate infrastructure, insufficient financial and social support, and a failure to address the psychological concerns of both patients and caregivers adequately.

• Additionally, there is inadequate training in palliative care, a significant shortage of human resources, and a scarcity of necessary medications. Furthermore, there is an absence of uniform guidelines supported by robust legal frameworks.

Implications of the practice, theory, or policy:

• Integration of health care services, curative and palliative, at all levels.

• Uniform policy/guidelines and robust legal support.

• Structures and systematic training programs for all health care professionals in end-of-life care.

• Patient and caregiver empowerment through awareness, advocacy, education, financial and psychosocial support.

**Supplementary Information:**

The online version contains supplementary material available at 10.1186/s12904-025-01825-z.

## Introduction

End-of-life care represents a critical dimension in the healthcare landscape, embodying the ethical imperative to ensure comfort, dignity, and compassion for individuals approaching the conclusion of their lives [1]. Globally, end-of-life care has been increasingly recognized as a critical area of healthcare, with countries like the UK, Australia, and Canada leading the way in establishing comprehensive palliative care services. As a nation characterized by cultural diversity, demographic nuances, and evolving healthcare dynamics, India stands at the intersection of tradition and modernity in shaping its approach to end-of-life care [2]. The growing elderly population and the increasing prevalence of chronic illnesses require understanding the healthcare infrastructure’s capacity to address the evolving needs of a diverse and aging society [2, 3]. Exploring the current literature allows us to discern patterns, gaps, and potential areas for improvement in the delivery of end-of-life care across different regions, socioeconomic strata, and healthcare settings [4]. 

Identifying gaps in policy and implementation provides a foundation for future research and policy development to enhance the quality and accessibility of end-of-life care services. An ethical imperative underscores end-of-life care to ensure a good quality of life and support system during a time marked by physical, emotional, and existential challenges [5]. 

Exploring end-of-life care in India within all its diversity is relevant to ensure it aligns with individuals’ and their families’ inherent values and expectations [6]. Applicability, in the context of this scoping review, extends beyond the theoretical realm to the practical considerations of healthcare delivery. While a body of literature exists on end-of-life care [7], a focused review of the Indian context is a novel undertaking. Still, it is also relevant to other countries, particularly Southeast Asia, which is culturally similar [8]. The cultural, religious, and social intricacies that characterize India contribute to a unique tapestry of experiences and challenges in end-of-life care [9]. 

This review is aimed to be a reference point for researchers, policymakers, and healthcare practitioners seeking to understand the current situation. It delves into the multifaceted landscape of end-of-life care in India, elucidating the existing literature and identifying the gaps, challenges, and opportunities that define the current situation and serve as a catalyst for future research endeavors, directing attention to areas where further investigation is warranted.

## Review question

How is end-of-life care accessed, provided, and supported, and what are its outcomes known in India?

### Objectives

The specific objectives of this scoping review were to examine the following dimensions of end-of-life care in India:

Access to care – the availability and reach of end-of-life services across geographic and demographic settings.

Capacity to provide care – the infrastructure, workforce, and system readiness to deliver end-of-life care.

Processes of care delivery – the mechanisms, protocols, and clinical pathways involved in end-of-life care provision.

Barriers and facilitators – factors influencing the implementation and uptake of end-of-life care at institutional and community levels.

Outcomes of care – the quality of end-of-life care as reflected in patient and caregiver experiences, quality of death, symptom management, and psychosocial well-being.

## Methods

### Protocols and registration

The protocol was registered with Open Science Framework (OSF) on 29th November 2023 (https://osf.io/twc9j).

### Study design

This scoping review was conducted following the Joanna Briggs Institute (JBI) methodology for scoping reviews, which is well-suited to synthesize a broad range of evidence on a particular topic, especially where the area is under-researched [10]. The JBI framework is ideal for exploring the breadth of end-of-life care in India. It allows the identification of gaps in the literature and offers an inclusive review of different types of research (qualitative, quantitative, and mixed methods) on this topic [11]. 

The review is reported according to the Preferred Reporting Items for Systematic Reviews and Meta-Analyses extension for Scoping Reviews (PRISMA-ScR) guidelines, ensuring that the process was rigorously documented from the initial search to data extraction and analysis [12]. 

### Eligibility criteria

Inclusion and exclusion criteria were established to ensure only relevant studies were reviewed (Table 1).

**Table 1:** Inclusion and exclusion criteria

**Inclusion Criteria**	
Studies that aim to assess the accessibility, availability, stakeholder perspective, and outcome of end-of-life care in India. Studies focused on the population living within India. Empirical research published in the English language. Studies published from January 1990 till 31st May 2024	
**Exclusion Criteria**	
Studies focused on euthanasia and single case-based papers. Opinion papers, newspaper articles, review articles, editorials, court orders, and policy statements. Studies related to pediatric end-of-life care (aged less than 18 years) due to its distinct nature in care delivery, ethical considerations, and family dynamics, which merit a separate in-depth review	

### Search strategy

To comprehensively identify relevant studies, we employed a three-step search strategy recommended by the Joanna Briggs Institute [11–13]. First, an initial limited search of two key databases, MEDLINE and SCOPUS, was conducted to identify appropriate search terms, including Medical Subject Headings (MeSH) and keywords related to end-of-life care (e.g., “end-of-life care,” “palliative care,” “hospice,” “caregiver,” “India”). This initial search helped refine and finalize the terms used for broader database searches. (Supplementary-1: Search Strategy)

In the second step, the search strategy was expanded to include the following databases: Web of Science, CINAHL, EMBASE, and PsycINFO, ensuring a comprehensive literature search covering a range of disciplines relevant to end-of-life care. The search covered the period from January 1, 1990, to May 31, 2024. The search terms were applied to titles, abstracts, and keywords, and Boolean operators were used to optimize search sensitivity (e.g., “AND,” “OR,” and “NOT”).

The third step involved manual searches of the included studies’ reference lists and grey literature sources such as Google Scholar, Open Grey, conference proceedings, and clinical trial registries. Additionally, full-text searches of relevant papers were performed, and where necessary, corresponding authors were contacted to obtain full-text articles for studies that were otherwise inaccessible.

### Screening and study selection

Two independent reviewers (BV and AG) used Rayyan data management software to screen the research papers’ titles and abstracts against the eligibility criteria [14]. They also assessed relevant full-text articles, and any differing opinions or areas of uncertainty were resolved by joint discussion with team members (CM and NS).

### Data extraction

Data extraction was conducted using a custom-designed data extraction tool based on the JBI template for the source of evidence details and characteristics [13]. This tool allowed for the systematic collection of data across multiple dimensions of end-of-life care. The data extraction tool was updated to reflect the revised objectives, including care outcomes as a distinct domain. Extracted outcome-related variables included indicators of quality of death, symptom burden, emotional and psychological well-being, caregiver distress, and social impact. Legal and policy frameworks were also documented to inform structural factors influencing care delivery. The following data were extracted from each included study (Supplementary-2: Data Charting Tool).

#### General study information

Title, author(s), year of publication, journal/source, and country of origin.

#### Study characteristics

Study design, methodology (qualitative, quantitative, or mixed), and population/sample characteristics.

#### Key findings

Relevant insights on end-of-life care, including barriers, facilitators, stakeholder perspectives, legal and policy frameworks, and outcomes.

#### Documented identified gaps

Areas within the publications where additional research is required were documented.

Two reviewers independently performed the data extraction process and cross-checked it to ensure accuracy. A third reviewer resolved any discrepancies through consensus.

### Data synthesis

The extracted data were thematically organized into five key areas: availability and access to end-of-life care, psycho-social aspects of care, barriers and facilitators to care provision, quality of end-of-life care and death, and education and training of healthcare providers. Descriptive statistics were used to summarize the distribution of study types, methodologies, and key characteristics, while qualitative synthesis was used to identify patterns and themes across the studies. Thematic synthesis allowed for the aggregation of findings across multiple studies, helping to draw out key insights into the current state of end-of-life care in India. After the data extraction, the reviewer (BA) categorized the studies into various themes based on the findings and finalized them after a discussion with the supervisors (CRM and NS). The reviewed data was arranged according to the final themes.

We employed a thematic synthesis approach to enhance analytical depth, consistent with the scoping review methodology as outlined in the Joanna Briggs Institute Manual for Evidence Synthesis [15]. Although our predefined objectives guided the initial data extraction, the thematic classification was developed iteratively during the review process.

Following Braun and Clarke’s thematic analysis framework, we conducted multiple readings of the extracted data to identify recurrent patterns and concepts across studies. Emerging themes were refined through iterative discussion among the review team (BA, CRM, NS), ensuring they reflected the diversity of data sources, contexts, and study populations [16]. Themes were then mapped against the original review objectives to ensure conceptual coherence and to capture the multidimensionality of end-of-life care in India.

This process enabled us to organize findings into five key themes: (1) Quality of end-of-life care and death, (2) Availability and access, (3) Psycho-social aspects, (4) Barriers and facilitators, and (5) Education and training. These thematic domains reflect inductively derived categories and deductive alignment with the study’s objectives, thus strengthening the analytic validity of the synthesis.

Results were presented following the PRISMA-ScR guidelines, ensuring transparency in the screening and selection process reporting.

## Results

### Study selection and characteristics

**Fig. 1:**
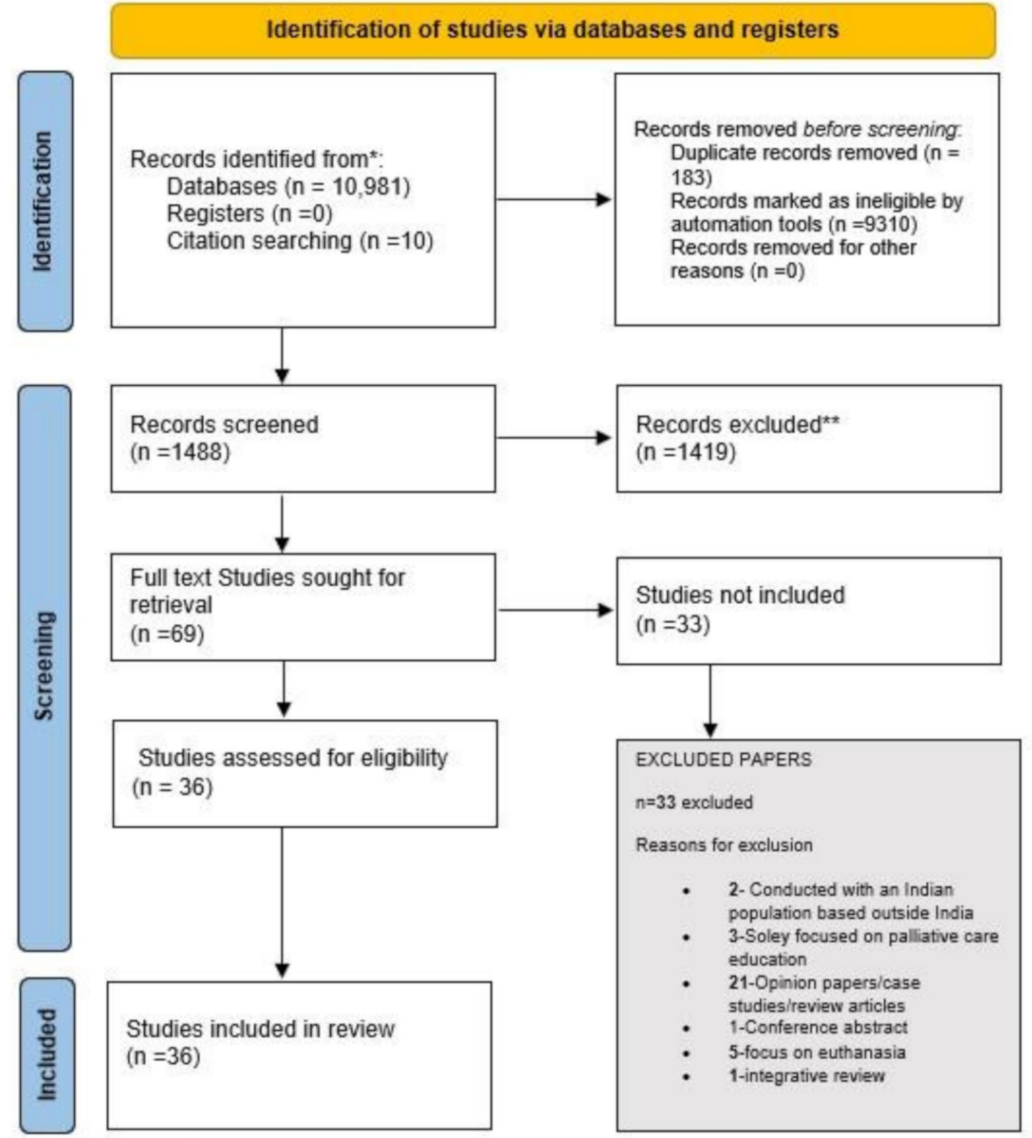
PRISMA ScR flowchart *Consider, if feasible reporting the the number of records identified from each database or register searched (rather than the total number of across all databases/ registers) **If automation tools were used, indicate how many records were excluded by a human and how many were excluded by automation tools *From*: Page MJ, McKenzie JE, Bossuyt PM, Boutron I, Hoffmann TC, Mulrow CD, et al. The PRISMA 2020 statement: an updated guideline for reporting systematic reviews. BMJ 2021;372:n71. doi: 10.1136/bmj.n71

Of the 1488 studies initially identified, 69 full papers were screened, and 36 were included in the review. (Fig. 1 PRISMA-Scr flowchart). Details of the included studies are in Supplementary−3: Overview of included studies.

### Range of studies

The types of studies included in the review are mentioned in Table 2.

**Table 2:** Types of studies included

Type of Study	Number
Qualitative study	15
Mixed Methods	03
Survey	09
Prospective Data Review	01
Retrospective study (data/chart review)	06
Pre & post-test	02

Eleven studies focused on the perspectives of primary caregivers and patients on end-of-life care [17–28], four on both healthcare professionals’ and primary caregivers’ views [29–32], and three papers looked exclusively at the perspectives of professional stakeholders [33–35]. 

Nine papers examined the attitude, awareness, availability, experience, and impact of end-of-life care decisions in tertiary care centers or Intensive care units [36–44]. 

Four papers looked at the importance of training, education, and implementation of end-of-life care plans in competency building for professional caregivers- clinicians, intensivists, oncologists, and nurses to improve the quality of end-of-life care provided in tertiary care, intensive care units, and general hospitals [45–48]. One paper examined the quality of death and dying and where India stands compared to other countries [49]. 

Two studies examined the cross-cultural comparison of palliative and end-of-life care between India and other countries [50, 51]. One study gave the profile of patients and the cause of admissions to hospices [52], and one study looked at the high economic cost of end-of-life care and dying in India [53]. 

### Overview and thematic alignment with objectives

The results have been organized thematically to ensure a coherent synthesis of findings. These themes were developed iteratively during data extraction and analysis in alignment with the review’s predefined objectives:

- Access to Care.
- Capacity to Provide Care.
- Processes of Care Delivery.
- Barriers and Facilitators to Care Provision.
- Outcomes of End-of-Life Care.

**Fig. 2:**
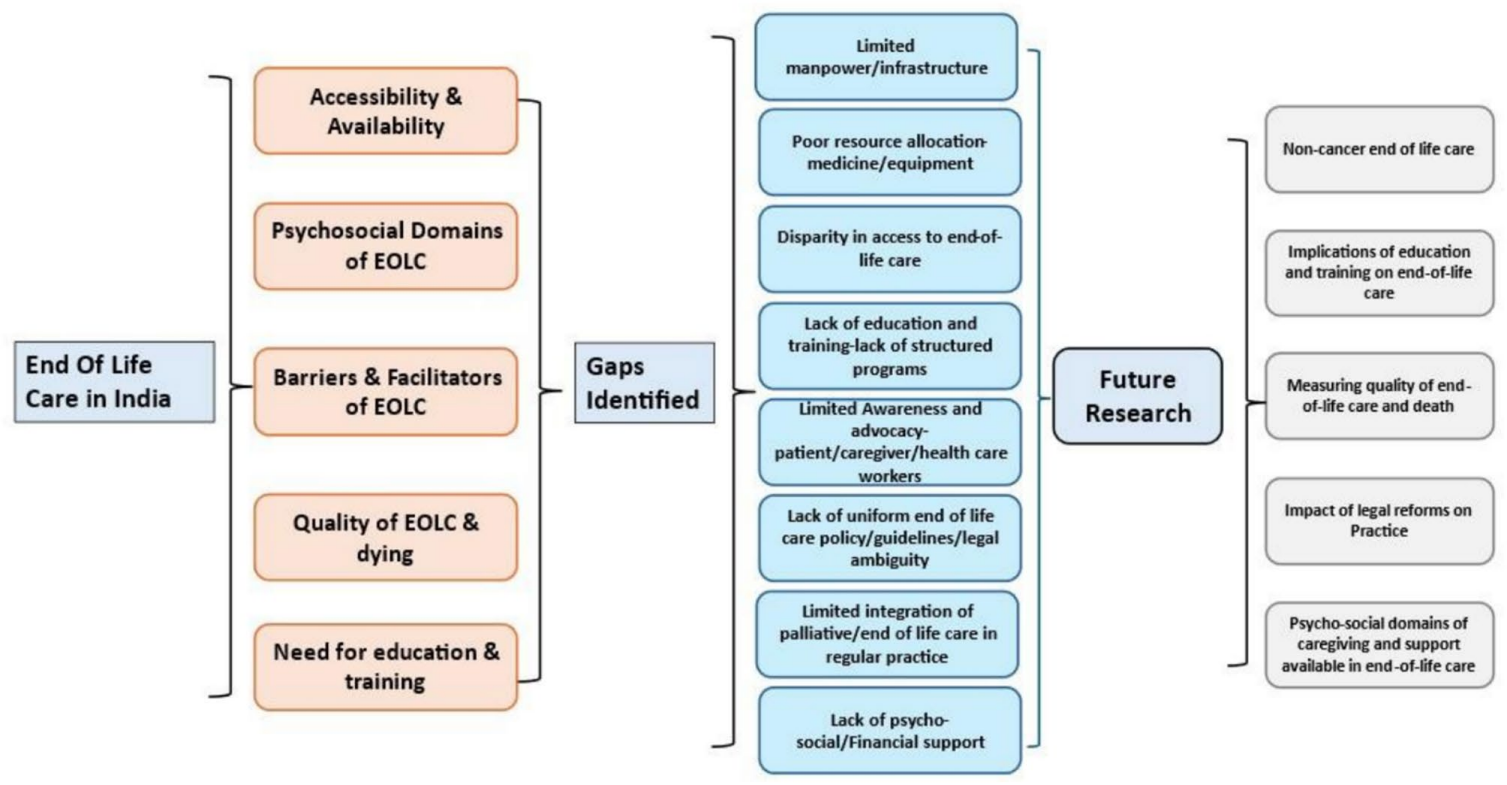
End of life care framework

Thematic synthesis allowed us to explore the complexities of end-of-life care in India while ensuring that each theme aligns with one or more of our review objectives. This synthesis identified five major themes related to end-of-life care in India: availability and access, psycho-social aspects of care, barriers and facilitators, quality of care and death, and education and training for healthcare providers. These themes collectively highlight the critical aspects of effective end-of-life care in the region. (Figure-2 Thematic framework of mapping end-of-life care in India)

### Theme 1: Quality of end-of-life care and death

Studies revealed that India ranks poorly in global assessments of the quality of death and dying, with inadequate symptom management, poor communication regarding prognosis, and the absence of formal end-of-life care protocols in many healthcare settings [33, 49]. India was rated 59th out of 81 countries in a global assessment that used indicators such as recognition of palliative care, availability of opioids, and national laws/guidelines on palliative care [49]. 

Additional issues included a lack of sensitive communication when conveying diagnosis and prognosis, incomplete knowledge about end-of-life care and its components, and the unavailability of end-of-life care protocols in hospitals [32, 43]. 

The quality of death was measured by the physical discomfort, symptom burden, psychological discomfort- fear of death/existential distress, prognostic awareness- caregivers practicing collusion [19], uncertainty regarding disease state, perceived burden, and negative emotions- caregiver fatigue, frustration, and exhaustion at the time of death [25, 50]. Patients who received hospice or home-based palliative care generally reported better symptom relief and emotional support than those who received care in hospitals, particularly intensive care units [17, 25]. 

Several studies emphasized the need for standardized protocols to improve the quality of end-of-life care. Lack of clear guidelines and lack of caregiver awareness often led to unnecessary, aggressive treatments at the end of life, with patients frequently being subjected to non-beneficial interventions or delayed initiation of end-of-life care [37, 38]. Studies also noted that the quality of death was influenced by the availability of psycho-social support, with patients who had strong family or community support structures experiencing better outcomes [29]. 

### Theme 2: Availability and access to end-of-life care

End-of-life care services in India are largely fragmented, limited across all care settings, and especially sparse in rural areas and for non-cancer populations [17, 22, 32, 42]. The availability and access to palliative care services are underdeveloped; patients are often referred late, and there is limited access and availability of essential medicines like morphine [29]. In contrast, the region of Kerala stood out as an exception, with access to primary palliative care through community networks and government partnerships with non-profit organizations [50]. Evidence on hospital-based end-of-life care is mainly drawn from intensive care unit experiences, for example, limiting potentially inappropriate interventions at the end of life, withdrawal, and withholding of life-sustaining measures when the poor prognosis is documented [40, 42, 44]. 

Hospice-based end-of-life care is only available in a few parts of the country, is focused mainly on those with advanced cancer, and there are issues in terms of clinicians often being unaware of the hospice facilities.Click or tap here to enter text [32, 39]. 

Home-based palliative care is mainly nurse-led and seldom able to provide regular support, with care often fragmented or uncoordinated [26]. The doctor visits are infrequent and often do not adequately address patient and caregiver distress. Patient and caregiver views about their satisfaction with services varied.Click or tap here to enter text [20, 21, 33]. 

A few contextual studies included in this review highlight the poor quality of ageing and dying for patients receiving home-based Palliative care and suggest that home-based care in its present form may not be adequate to provide good-quality end-of-life care [21, 26, 29]. Social aspects of care like inappropriate housing, financial debility, and lack of safety with instances of elderly abuse at home due to caregiver burnout diminish the success of home-based end-of-life care [26, 29]. Two studies showed that although the chosen place of end-of-life care was home [28], on a few occasions, home-based services could not adequately manage symptoms, and hospital admissions were required [50]. The review found few established guidelines or referral pathways for transitioning patients from curative to palliative care, contributing to inconsistent care practices nationwide.

### Theme 3: Psycho-Social aspects of end-of-life care

Psycho-social challenges were a predominant issue. Factors associated with poor end-of-life care were lack of infrastructure, housing, safe place for care, financial distress due to loss of job or income (especially if the patient is the primary wage earner of the family, loss of identity, sense of being a burden to family, isolation from the society due to sickness and psychological and emotional burden of the disease [18].

Financial distress was a recurring issue, particularly for families caring for terminally ill patients in rural areas, where healthcare costs often led to impoverishment [31, 32, 42, 50]. Since end-of-life care is not covered under health insurance or through government health schemes, most terminally ill patients and their families struggle to provide good care [31]. A study on the inpatient care and cost of dying clearly outlined that the inpatient care cost of decedents was significantly higher than that of survivors. This was especially the situation for those who stayed longer, were from rural areas, were in private health care, or suffered from serious illnesses. Most expenditures were out of pocket, which led to severe financial liabilities [53]. 

Societal stigma, especially where cancer is concerned, the burdens of caregiving may lead to social isolation, psychological distress, and physical exhaustion [50]. Terminally ill patients are mostly cared for at home, and despite the psychological support provided, a significant number of patients and their families were found to be very anxious [20]. Many families were forced to rely on informal caregiving structures with limited or no professional support. This lack of support led to a high caregiver burden, particularly for women, who were often expected to take on most caregiving responsibilities due to traditional gender roles [50]. 

Furthermore, cultural practices such as “Thalaikoothal” in some rural areas reflected families’ extreme socio-economic pressures, where the lack of accessible, affordable care forces them into desperate measures [27]. Thalaikoothal, or the traditional practice of senicide (killing of the sick and debilitated elderly), is perceived as freeing the terminally ill elderly patient from painful death and is usually prevalent in rural areas of South India and culturally acceptable. It is practiced due to expensive hospitalizations, inadequate hospital care, dying patients discharged home to die, financial constraints of the family, restricted social interactions due to time spent caring for the sick patients at home, and lack of proper access to palliative care [27]. 

### Theme 4: Barriers and facilitators to care

#### Barriers

One of the most prominent barriers to end-of-life care is the lack of awareness and understanding among healthcare professionals and the public due to inadequate awareness, education, and training [29, 35, 36, 49]. Many studies pointed to the discomfort healthcare providers felt in discussing death and dying, leading to poor communication with patients and families about end-of-life decisions [33, 45].

Legal barriers were also frequently cited, particularly regarding withdrawing life-sustaining treatment and using advance directives [42]. Although the 2024 Supreme Court ruling allowing advance medical directives is a significant step forward, its impact on clinical practice remains limited due to a lack of awareness and inconsistent implementation across healthcare institutions [33, 54]. One identified main barrier was the lack of robust laws and legal systems for end-of-life care and the lack of hospital policy [33]. Legal ambiguity can cause aggressive management or discharge against medical advice. Documentation of poor prognosis was the main factor associated with end-of-life decisions in tertiary care centers [44]. Withdrawal or withholding of care, although practiced within some intensive care units, is still not the norm [42]. The lack of clear legal guidelines and the absence of advance directives make it difficult to make end-of-life decisions [36, 38]. 

Other barriers included the unavailability of essential palliative care medications, such as opioids, due to regulatory hurdles and a shortage of trained palliative care professionals [29, 32, 33]. 

### Facilitators

The identified leading facilitator for providing end-of-life care was the presence of experienced healthcare providers in urban tertiary care hospitals, who were more likely to engage in appropriate discussions and provide patient-centered care [43].

Kerala’s successful integration of community volunteers into palliative care services was also seen as a model for other regions. Availability of care through home care teams, community support through volunteers and community networks, low-cost morphine, specialized palliative care services at a few centers, and government funding [29, 50] were major facilitating factors that contributed to this model’s success.

The review found that as early as 2009, intensive care units were one of the spaces where end-of-life decisions were being taken. Some of the most common reasons are the advanced nature of the disease, unresponsiveness to treatment, severe neurological deficits, and family decision to discontinue treatment, which ultimately reduced the therapeutic interventions in such patients but were seen to have a longer stay in intensive care units [42]. 

### Theme 5: Education and training in end-of-life care

A critical gap was the lack of education and training in end-of-life care for healthcare professionals, which led to a lack of competency and discomfort in discussing end-of-life care issues with patients and primary caregivers [31, 36]. 

Compared with other specialities, those with a background in internal medicine or critical medicine were perceived as better able to care for patients with end-of-life care needs [33, 40]. For example, senior clinicians, intensivists, and experienced intensive care unit nurses could recognize the dying process, initiate end-of-life conversations, and facilitate decision-making about appropriate goals of care (avoiding aggressive interventions) [41, 43]. 

Integrating end-of-life care into the medical and nursing curricula was seen as a core component to ensure that future healthcare professionals are equipped to handle the complexities of end-of-life situations [30, 41, 45, 47]. 

For example, including end-of-life care as a key module within the training curriculum for doctors and nurses in their education programs, promoting Compassionate Communities, and higher investments in research are seen as ways to improve end-of-life care [36, 43, 49]. 

Training programs such as the ‘End-of-Life Nursing Education Consortium’ and stakeholder engagement in intensive care units are proposed to improve palliative care knowledge and define the concept of good death, end-of-life care practices, and attitude towards care of the dying [45–47]. 

Empowering the caregiver by providing adequate support and training for caregiving is needed, especially for those who are caring for terminally ill patients at home [17, 26, 29, 50]. 

## Discussion

End-of-life care in India is characterized by significant fragmentation and inconsistent provision. While there have been promising developments in urban areas and regions such as Kerala, many people, especially those in rural or underserved areas, remain without access to appropriate end-of-life care.

One of the most pressing issues is the disparity in access to end-of-life care services, particularly for non-cancer patients and those living in rural areas [55, 56]. This inequity is largely attributed to infrastructural deficiencies, limited numbers of trained professionals, and low public and professional awareness of palliative care. This has resulted in inadequate care for many patients at the end of life, which reflects broader issues within India’s healthcare system, where access to specialized care is often limited to urban centers [57]. Efforts to expand community-based palliative care services, as seen in Kerala, offer a promising model for other states to follow [58]. Integrating palliative care into primary healthcare settings and increasing government support for community-based initiatives could help address the geographic and economic barriers to care provision [59]. Moreover, establishing clear referral pathways for transitioning patients from curative to palliative care, especially for chronic non-cancer conditions, would ensure that more patients benefit from timely end-of-life care interventions [60]. 

Cultural factors play a significant role in shaping attitudes toward death and dying in India. The review highlighted the socio-economic and cultural pressures that often lead to inadequate or inappropriate care at the end of life. Practices such as “Thalaikoothal” reflect the desperation of families who lack access to formal healthcare services [27]. Addressing these challenges requires a nuanced approach, considering the socio-cultural context while promoting humane and compassionate care alternatives [61]. 

### What this study adds

End-of-life care in India faces significant challenges, including limited resources, urban-rural disparities, and socio-cultural influences that affect care delivery and attitudes towards death. Existing research indicates a lack of access to specialized palliative care, legal uncertainties surrounding end-of-life decisions, and insufficient integrated services, particularly for chronic non-cancer conditions. The evidence base is often inconsistent and primarily focuses on advanced cancer care in urban tertiary settings, neglecting non-cancer populations and primary care contexts. Efforts to expand community-based palliative care, such as the Kerala model, highlight the potential for improvement, though these initiatives require adaptation to local conditions. A cautious approach to policy recommendations is necessary due to the limited quality of existing evidence; instead, a targeted research agenda should prioritize longitudinal studies, evaluation of legal reforms like the 2024 Supreme Court ruling on advance directives, and deeper exploration of the psycho-social aspects of caregiving [62]. Additionally, cultural and socioeconomic factors, such as the practice of Thalaikoothal, underscore the complexities of integrating palliative care with family resources and societal expectations. This review maps the availability, delivery processes, and outcomes of end-of-life care in India, calling for robust research that reflects the country’s diversity and emphasizes the integration of palliative care into primary healthcare, especially in rural areas, while advocating for greater awareness and standardization of practices across healthcare institutions. To bridge the gaps identified, literary evidence suggests the following actions for clinical practice and policy, including:

#### Policy and training

There is an urgent need for widespread education and training in end-of-life care for healthcare providers [63, 64]. Initiatives such as the End-of-Life Care Nursing Education Consortium have demonstrated that targeted training can significantly improve clinicians’ and nurses’ ability to provide compassionate care [46, 64]. To ensure long-term improvements, end-of-life care must be integrated into the medical and nursing curricula nationwide [46, 65]. Efforts must be made so that end-of-life care policies align with legal reforms, widespread education initiatives targeting healthcare professionals and the public, and include palliative care in medical and nursing curricula.

#### Community-based care

Expanding community-based palliative programs that are physician- or nurse-led and supported by local neighborhoods, especially in underserved regions [64]. 

#### Future research

Further studies should explore integrating palliative and curative care for non-cancer patients, investigate the psycho-social dimensions of caregiving, and evaluate the impact of recent legal reforms on clinical practice.

### Strengths and weaknesses/limitations of the study

#### Strengths

This review maps the entire spectrum of end-of-life care in India, covering accessibility, psycho-social support, education and training, and quality of care. It comprehensively synthesizes evidence across multiple domains and identifies critical gaps in care provision, particularly for non-cancer patients and rural populations.

#### Limitations

This review has several inherent limitations, primarily due to the nature of scoping reviews. Hand-searching of key journals was not conducted, and specific searches using terms like “palliative care” or “euthanasia” were omitted. The inclusion of only English-language publications and a focus on studies from the Indian subcontinent may have led to the oversight of some relevant data sources. Additionally, a quality appraisal of the included studies was not performed (as such evaluations are generally not recommended according to JBI guidance), limiting our ability to assess the methodological robustness of individual studies. Most of the research concentrated on advanced cancer or terminally ill cancer patients, with minimal coverage of non-oncology end-of-life care. The geographical focus on states with better-established palliative care systems further risks underrepresenting insights from regions with fewer resources. Lastly, while thematic synthesis helped to organize the findings, the diversity of methodologies and reporting styles across studies posed challenges in ensuring consistency and depth in the analysis.

### Future research directions

The review identifies the need for further exploration into several areas, including:

Integration of curative and palliative care: To enhance the continuity and quality of life and provide a seamless transition from curative to palliative.

Psycho-social aspects of caregiving: Investigating gender dynamics and the financial impact on caregivers, which could inform targeted interventions.

Legal and Policy Impacts: To examine how recent policy changes affect the clinical practice and patient outcomes in end-of-life care across diverse settings in India.

## Conclusion

This scoping review offers a comprehensive overview of end-of-life care in India, revealing major gaps in access, quality, training, and support, particularly for non-cancer populations and rural communities. It highlights the fragmented nature of existing research and the need for standardization, legal clarity, and community-based models tailored to local needs. The findings should be a reference point for researchers, educators, and policymakers to initiate further empirical work and capacity-building efforts. A multifaceted, evidence-informed, and contextually responsive strategy is essential to strengthen EoLC across India.

## Electronic supplementary material

Below is the link to the electronic supplementary material.

Supplementary Material 1

Supplementary Material 2

Supplementary Material 3

## Data Availability

Data is provided within the manuscript or supplementary information files.

## Abbreviations

EoLC: End-of-Life Care
PC: Palliative Care
ICU: Intensive Care Unit
JBI: Joanna Briggs Institute
PRISMA-ScR: Preferred Reporting Items for Systematic Reviews and Meta-Analyses extension for Scoping Reviews
OSF: Open Science Framework
CINAHL: Cumulative Index to Nursing and Allied Health Literature
EMBASE: Excerpta Medica Database
MeSH: Medical Subject Headings
ELNEC: End-of-Life Nursing Education Consortium
SCC: Supreme Court of India (used in the context of legal judgment reference)
KIPCER: Karunashraya Institute for Palliative Care Education and Research
