# Supplementary material for: Mapping end-of-life care in India: a scoping review to identify gaps in policy, practice, and psychosocial support
Source: BMC Palliat Care. 2025 Jul 7;24:189. doi: 10.1186/s12904-025-01825-z (PMC12235897; doi:10.1186/s12904-025-01825-z)
Supplement: Supplementary file 1 — Supplementary Material 1 [file 12904_2025_1825_MOESM1_ESM.docx]

**Supplementary-1**

**Search Strategy**

(Language: English; adult; peer-reviewed articles only)

**Scopus**

TITLE-ABS-KEY ( "end of life care" OR " terminal care" OR palliati* OR hospice ) AND ORIG-LOAD-DATE AFT 20240520 AND ( LIMIT-TO ( AFFILCOUNTRY , "India" ) ) AND ( LIMIT-TO ( SUBJAREA , "MEDI" ) OR LIMIT-TO ( SUBJAREA , "PHAR" ) OR LIMIT-TO ( SUBJAREA , "NURS" ) OR LIMIT-TO ( SUBJAREA , "NEUR" ) OR LIMIT-TO ( SUBJAREA , "SOCI" ) OR LIMIT-TO ( SUBJAREA , "IMMU" ) OR LIMIT-TO ( SUBJAREA , "PSYC" ) OR LIMIT-TO ( SUBJAREA , "HEAL" ) OR LIMIT-TO ( SUBJAREA , "MULT" ) ) AND ( LIMIT-TO ( LANGUAGE , "English" ) )

**Medline & PubMed**

"Palliative Care"[Mesh] OR "Hospice and Palliative Care Nursing"[Mesh] OR "Palliative Medicine"[Mesh] OR "Terminal Care"[Mesh] OR "Hospice Care"[Mesh] OR ("end of life care") OR (EOLC) OR terminally ill[MeSH] OR ("terminal patient*") OR ("life-limiting illness") OR ("life-limiting condition*") OR ("terminal phase") OR ("terminal stage") OR ("EOL care") OR ("comfort care") OR ("hospice program") OR (hospice*) OR hospice[MeSH] OR ("supportive care") OR ("supportive treatment") OR ("supportive therapy") OR ("bereavement care") OR ("bereavement counselling") OR ("symptom management") OR ("symptomatic treatment") OR ("symptomatic therapy") OR ("advanced illness") OR (palliat*) OR ("end of life")

India:

"India"[Mesh]OR India*[tiab]OR Indian*[tiab]OR Indian[PR]OR India[ad]

Life limiting diseases:

("cancer") [Mesh] OR ("congestive cardiac failure") [Mesh] OR "CCF" [Mesh] OR "chronic obstructive pulmonary disease” [Mesh] OR "COPD" [Mesh] OR "neuromuscular disorders" [Mesh] OR "chronic liver failure" [Mesh] OR "CHF" [Mesh] OR "chronic renal failure"[Mesh] OR "CRF" [Mesh] OR "stroke" [Mesh] OR "multiple sclerosis" [Mesh] OR "Parkinson’s disease" [Mesh] OR "rheumatoid arthritis" [Mesh] OR "neurological disease" [Mesh] OR "dementia" [Mesh] OR "congenital anomalies" [Mesh] OR "AIDS" [Mesh] OR "HIV" [Mesh] OR "brain injury" [Mesh] OR "spinal cord injury" [Mesh] OR "motor neuron disease" [Mesh] OR "genetic disorders" [Mesh] OR "connective tissue disorders" [Mesh] OR "life limiting diseases" [Mesh] OR "malignancy" [Mesh]

*Participant: (additional terms )

"Caregiver perspectives" [Mesh] OR "Terminally ill Patient"[Mesh]
