# Supplementary material for: Mapping end-of-life care in India: a scoping review to identify gaps in policy, practice, and psychosocial support
Source: BMC Palliat Care. 2025 Jul 7;24:189. doi: 10.1186/s12904-025-01825-z (PMC12235897; doi:10.1186/s12904-025-01825-z)
Supplement: Supplementary file 2 — Supplementary Material 2 [file 12904_2025_1825_MOESM2_ESM.docx]

**Supplementary-2: Data Charting Tool**

| **Scoping Review Details** | |
| --- | --- |
| Scoping Review title: |  |
| Review objective/s: |  |
| Review question/s: |  |
| **Inclusion/Exclusion Criteria** | |
| Population |  |
| Concept |  |
| Context |  |
| Type of evidence/paper |  |
| **Evidence Source Details and Characteristics** | |
| Citation details (e.g., author/s, date, title, journal, volume, issue, pages) |  |
| Context |  |
| Participants |  |
| Data/Results extracted, including legal and policy frameworks,  psychosocial outcomes, and end-of-life outcomes | |
| Gaps identified |  |
| Limitations of the study |  |
