# Supplementary material for: Mapping end-of-life care in India: a scoping review to identify gaps in policy, practice, and psychosocial support
Source: BMC Palliat Care. 2025 Jul 7;24:189. doi: 10.1186/s12904-025-01825-z (PMC12235897; doi:10.1186/s12904-025-01825-z)
Supplement: Supplementary file 3 — Supplementary Material 3 [file 12904_2025_1825_MOESM3_ESM.docx]

**Supplementary-3: Overview of included studies**

| **Sl No** | **Title of Study, Author, Year, and place of origin** | **Type of Study** | **Aims** | **Study Population** | **Key findings** | **Gaps identified** |
| --- | --- | --- | --- | --- | --- | --- |
| 1 | Limitation and withdrawal of intensive therapy at the end of life: Practices in intensive care units in Mumbai, India,  Farhad Kapadia; Manoj Singh; Jigeeshu Divatia; Vaidyanathan Priya; Farokh E. Udwadia; Sumit J. Raisinghaney; Harshad S. Limaye; Dilip R. Karnad, 2005, India | Review of prospectively collected data | This study aims to describe the practices at end of life in ICUs' in Mumbai hospitals with regard to limitation and withdrawal of care. | Patients from four hospitals who died in the hospital | The study showed that of the 1045 deaths 27%(143) occurred in ICU. Of the total death in ICU 34 % had limitation of therapy terminally. This was mostly seen in Cancer hospital- 50 %. Most of terminal intensive care facility offered in Private hospitals were to accommodate the relatives of the patient to avoid legal issues even though ICU care would have been futile. Where as a large number of patients in ICUs' in Public hospitals were young with out much co-morbidities. since the ICU space is limited in Public setups. Study states that limitations of the care may occur in Indian hospitals regardless of legal clarity on the subject. | Lack of legal support and clarity where withdrawal of support is considered with regards to End of life care in ICUs' Gaps highlight were the need for clearer legal guidelines, better resource allocation, and more comprehensive data collection to improve end-of-life care practices in Mumbai's ICUs. |
| 2 | End of life care of terminally ill geriatric patients in Northern India  Vivek Gupta, Sandeep Kumar, Abhishek Shukla, Shailendra Kumar, Surendra Kumar, 2007, India | Cross Sectional Qualitative Study | To study the knowledge attitude and practices of EOLC issues of terminally ill geriatric cancer patients | Doctors , Geriatric Cancer patients and Caregivers | 28% of Physicians could name 03 important elements of EOLC, 95% wanted to use specialists Palliative services but were unaware of such facilities. Need of specific Govt Health Scheme for Palliative Care Services | Lack of Physician Knowledge and Training: no physicians receive special training in end-of-life care during their undergraduate or postgraduate years.  Inadequate Use of Palliative Care Services: Most physicians sent terminally ill patients’ home under the care of local general practitioners, who felt inadequate in advising on terminal care and palliation.  Poor Communication and Counselling: Explanations and counselling provided by physicians were mostly inadequate and did not fully satisfy patients and their relatives.  Lack of Awareness Among Patients and Families: lack of awareness in patients and caregivers of the existence of hospices and alternative options Insufficient Infrastructure and Services: minimal support through NGO  Cultural and Regional Influences ack of information to patients about their diagnosis of advanced cancer, adding to the anxiety and helplessness of patients and their relatives.  Need for Policy and Educational Reforms: Lack of curriculum-based learning or organized teaching of end-of-life care issues. Health policymakers in India have not adequately addressed the quality of end-of-life care.  Financial and Logistical Challenges: for patient and families |
| 3 | Physician beliefs and practice regarding end-of-life  care in India,  V. Theodore Barnett, V. K. Aurora, 2008, India | Survey | Physicians beliefs and practices on withdrawal of life support in intensive care units | Physicians in a meeting in New Delhi (NAPCON) | The survey respondents were mostly males, 64% said that withdrawal of life was not supported and 35% said yes. 17 Physicians stated, though withdrawal was not allowed but it was still practiced. The legal issues were considered to be the biggest barrier in providing good EOLC in h hospital based settings. 92% of the respondents stated that the DNR was decided by the family and 5% said the patient decided. The study points out that withdrawal of life support was not used and if used it was mostly with the family and that these decisions should be combined between the medical team and the family of the patient. The study also found major barriers to good EOLC in India, as per physicians was mainly legal and administrative. It has nothing to do with ethical or cultural barriers. The EOLC perspective is also based on religion and culture and it is a major consideration for patients and their families. LAMA (Left against Medical Advice) is a common practice especially in India and is thought of as withdrawal of treatment by the patients family. it also looks at implicitly or imminence of death. In this study it was flaring obsecration that 59% of the phychians do not used DNR as compared to 6% of European Physicians. After this study was conducted a position statement looking at EOLC was made by Indian Society of Critical Care Medicine. | Legal and administrative barriers to EOLC, need for clarity and understanding regarding EOLC between patients, families and medical teams. Gaps indicated are the need for comprehensive legal and policy reforms, better communication and decision-making frameworks involving patients and families, and increased awareness and training for physicians regarding end-of-life care practices. |
| 4 | End of life care decisions in an Indian intensive care unit,  Raj Kumar Mani, Amit Kumar Mandal, Sabyasachi Bal, Yash Javeri, Rakesh Kumar, Deepak Kumar Nama, Praveen Pandey, Tara Rawat, Navneet Singh, Hemanth Tewari, Rajiv Uttam, 2009, India | Retrospective Study (Observational) | Document the end of life decisions and describe the process of decision making among patient dying in ICU | Patients admitted between May 2006 to Dec 2007 who died in ICU (12 bedded closed medical surgical ICU- Consecutive patients admitted) | No significant difference in the age or sex distribution between EOLD and FS groups. More co-morbidity in EOLD group. Longer ICU stay in EOLD. Study showed that EOLD significantly reduced the therapeutic and financial burdens in the last 3 days of life. Futility of care, failure of prolonged trial of ICU, poor premorbid functional status, poor long term prognosis, advanced age, financial burden and family's request were some of the major decisions for EOLD | Gaps identified are lack of more comprehensive data, clearer guidelines, education for healthcare providers, and inconsistent practices across different healthcare settings in India. |
| 5 | Learning from each other: cross-cultural insights on palliative care in Indian and Australian regions.  Pam McGrath, Hamish Holewa and Thomas Koilparampil, Cherian Koshy, Shobha George, 2009, India & Australia | Descriptive Phenomenology | The aim of the research was to explore the differences and similarities between Palliative care services in Kerala and South-east Queensland | Health care professionals and patients | 1.Palliative care principles and practice- volunteers provide financial support , infrequent training once a month in Palliative care.  2.Talking about death: only caregivers are aware, patients are not aware about their prognoses so unable to prepare for death, asked to stay in hospital in last days(no preferred place of death).Patients referred to palliative care without knowing that they are dying. Oncologists may not tell the patient about their terminal stage and many end up dying in Curative care. Unable to complete treatment due to poor financial status. These are the signifanct differences in Indian system as compared to Australia.   Mutual learning from each other may benefit the palliative care services provision. It will improve the local services ."Firstly, there was an understanding of the significance of honesty in information-giving to the patient. Secondly, the importance of palliative care specialists providing education to mainstream health professionals was recognized. Thirdly, the need for palliative care to be cognizant of the socio-economic impact of dying, especially for families experiencing poverty, by embracing strategies for financial and material support, was appreciated" | Gaps highlight were the need for improved communication, financial support, professional education, and accessible pain relief in palliative care services to better meet the needs of patients and their families at the end of life. |
| 6 | Care of Terminally Ill Cancer Patients: An Intensivist's Dilemma,  Sukhminder Jit Singh Bajwa, Sukhwinder Kaur Bajwa, Jasbir Kaur, 2010, India | Retrospective Study | Evaluate the benefits of Intensive care treatments in terminally ill cancer patients and whether optimal utilization of critical care resources has any positive financial, psychological and clinical outcome | Terminally Ill cancer patients admitted to ICU from 20th Dec 2006 to 20th April 2010. | The results showed 64.15% required airway protection and were subsequently put of mechanical ventilation. The statistical analysis was significant with p value less than 0.05. 79.24% required haemodynamic stability, mortality was significantly was higher in these patients (77.36% and 42 patients out of 53 developed multiorgan failure and expired).  Criteria of ICU admission should be clearly defined in such patients, alternative facilities to ICU admission should be evaluated and recommended for such patients thus relieving the economic burden on scarce resources. | Resource Allocation and financial constraints: Significant challenge in optimally utilizing ICU resources. . Prolonged ICU stays for terminally ill patients add to the economic burden on their families, who may already be financially depleted from ongoing cancer treatments Lack of Alternatives: There is a need for alternative care options, such as hospice care, which focuses on palliative rather than curative treatment.  Advance Directives and DNR Orders: The absence of clear guidelines and the reluctance to implement Do Not Resuscitate (DNR) orders complicate decision-making for healthcare providers.  Education and Awareness: There is a lack of proper education and awareness among the general population about the intricacies of intensive and palliative care, leading to unrealistic expectations from ICU admissions and interventions. |
| 7 | What does the informal care giver of a terminally ill cancer patient need? A study from a cancer centre,  Anjum S Khan Joad, T C Mayamol, Mohita Chaturvedi, 2011, India | Qualitative Study | To assess the needs of informal or family care givers of terminally ill cancer patients | Bereaved caregivers of cancer patients | Medical Domain- 82% was satisfied with the services of palliative care department whereas 17% were felt the lack of home care services and requested a dedicated home care service. 95% were prepared for an emergency. 92% of caregivers had no experience of caregiving out of that 71% felt need for training in caregiving. 40% of caregivers felt need for admission to in-patient facility.  Psychological Domain- 68% had no time for selfcare, 45% felt caregiving affected dynamics between friends and family. 45% complained of mental fatigue and irritability.  Financial Domain- 20% caregivers had financial difficulties, 25% felt reduced opportunities and lack of career advancement due to the additional caregiving role. 30% needed financial help from family and friends. Information Domain- 90% were aware about the patients condition and 96% were aware of all treatment options. Social Domain- 71% did not have time to socialize. 30% felt caregiving affected their relationships. Unmet Needs- 52 out of 56 caregivers felt need for more counselling. Caregivers experienced economic, physical, psychosocial burden as a result of caring for terminally ill patients. Many felt need for support for symptom management. The greatest needs were psychological, financial and information. | Need for good home care system as an addition to the in-patient facility. Support in caregiving via counselling and training. Financial support for caregiving through various resources.  Need to identify the vulnerable caregivers like younger age women and those caring for end stage patients. |
| 8 | Views on death and dying in an Indian cancer care hospice: Balancing individual and collective perspectives,   Carmen G Loiselle;Michelle M Sterling, 2011, India | Qualitative Study | This study aims to understand the experiences of various health care professionals in a hospice when confronted to dying and death of patients under their care | Health care professionals working at a hospice in Bangalore India(December 2008-July 2009) | The main themes that emerged were- 1. Paradox of death- a mixture of relief and sorrow, relief that suffering has ended and sorrow for the life lost.2.Balancing Individual with collective needs and well being- was based on the psychological support provide by the team members to each other. The effort by senior staff in providing sustained support to their juniors helped in reducing the distress among the nurses and auxiliary team. 3.Mindfullness of workplace initiatives as an exemplar of a thoughtfully designed grassroots initiative- workplace initiatives that supported the young nurses and nursing aides- like daily meetings to discuss their issues, progressive exposure to hospice work, team rapport and bonding.  The outcome was that there was a significant improvement in overall operations of training the nursing aides. | Young age of the nurses made it difficult for them to face death which are a constant in hospice settings. It also highlighted the importance of supportive measures like regular open dialogue within palliative settings to reduce staff distress and burnout due constant exposure to death and dying. Palliative care needs to increasingly pay attention to physical. psycho-social and functional needs of all stakeholders caring for terminal or dying patients |
| 9 | Psychosocial and spiritual problems of terminally ill patients in Kerala, India,  Elsner F, Schimdt J, Rajagopal M et al., 2012, India | Qualitative Study | To explore psychosocial and spiritual problems of terminally ill patients in Kerala. | Terminally ill patients visiting Trivandrum Institute of Palliative Sciences and Palliative Care Clinic in Trivandrum | Problems in emotional state, faith, family life, the study also found lack of knowledge about the disease and a major impact of financial issues.  To look at sources which can reduce the problems faced by terminally ill patients . A way to eliminate major issue would be through provision of sufficient funds since financial crisis is the main issue. The other problems related to tradition and culture need improvement. | Gaps identified were need for better communication skills and strategies, financial assistance, culturally sensitive care practices , emotional and psychological support |
| 10 | High Inpatient care cost of dying in India,   Laishram Ladusingh, Anamika Pandey, 2013, India | Retrospective Study on data published by national sample survey on morbidity and health care between 2004 and 2005 | To highlight the high cost of inpatient care of decedents in comparison to that of survivors in case of out of pocket health care cost in India | Hospitalized patients who died as compared to those who survived | The mean OOP expenditure of decedents is higher compared to survivors in all ages and OOP inpatient cost reduces with advancing age.   The Indian system of healthcare, the public system is unable to keep up with the high demand of provision and infrastructure of healthcare. As such it functions mostly on public-private partnership model, there is also no standardization or capping of fees based on medicines, equipment's facilities etc,. So both the outpatient and inpatient is exorbient in private sector. The facilities, infrastructure, equipments and amenities are of a better quality than the public system. Its also quite normal for economically strong patients to utilize private health care. One of the things observed was the expensive medications and cutting edge technology used in private sector which drive up the cost of medical care. The total non-medical expenses in OOP expenditure for I/P care is 8-12% irrespective of survival status or public-private affiliation. The OOP expenditure for females is less than that of the male patients which was statiscally significant. | Need for expansion of the public health care system Need for provision of adequate health care facilities for economically poor patients. To understand the health care needs of ageing population in India. |
| 11 | Preference of the Place of Death Among People of Pune  Priyadarshini Kulkarni, Pradeep Kulkarni, Vrushali Anavkar, Ravindra Ghooi, 2014, India | Survey | To study Preferred place of death in Population | Adults above 18 years of age residing in Pune Maharashtra | Preferred place of death-82.02 % at home,9.2%-elsewhere,7.7%hospital,females preferred home more than males, study reveals end of life preferences are not influenced by geo-political demarcation, economics or cultural differences.  Study was the first of its kind in India and highlighted the fact that human preferences may remain the same especially about the desired place of death. | Lack of Advanced Directives: In India, advanced directives are not commonly used or legally enforceable, limiting patients' ability to ensure their end-of-life wishes are followed.  Preference vs. Reality: there is often a discrepancy between this preference and the actual place of death due to various logistical and medical reasons.  Support for Home Deaths: There is a need for public and governmental policies to facilitate home deaths, including providing necessary medical support and equipment for terminal care at home.  Caregiver Burden: Family caregivers often feel unprepared to manage end-of-life care at home, indicating a need for better support and resources to help them.  Training and Awareness: There is a need for better training and awareness among healthcare providers and the general public about end-of-life care options and the importance of honouring patients' preferences. |
| 12 | Meaning of life experiences at the end of life; Validation of the Hindi version of the Schedule for Meaning in Life, Evaluation and a cross cultural comparison between Indian and German Palliative care patients  Dorothea Kudla , Julius Kujur, SJ, Sumanti Tigga, Prakash Tirkey, Puthita Rai, Martin Johannes Fegg, 2014, India& Germany | Cross Sectional Study | Aim was to provide a Hindi version of SMiLE and test its feasibility and validity in Indian Palliative care patients and compare the results with previous studies in Germany | Patients suffering from life limiting disease with life expectancy less than 6 months | Indian Palliative care patients listed spirituality significantly more often. Values and Partner were more important, and family work and social commitment were listed more often> Experience of end of life have comparable effects in both German and Indian Patients.  The study investigated MiL using in cross cultural and cross country population and did a comparative analysis. This study revealed significant culture specific differences with regard to social commitments and spirituality which was seen more in Indian group of patients. The study underlines the fact that to develop PC in India, economic support, family structure which are sustainable are important from patient perspective. The SMiLE tool can be used as a screening tool to identify patients in need. | Need for economic support, most patients are from very poor socio-economic background, need to build sustainable social support system for patients in need of PC. Gaps indicated are significant need for the development and expansion of palliative care services, better resource allocation, and increased awareness and education to improve end-of-life care in India |
| 13 | End of life care perspectives of patients and health care professionals in an Indian Health care setting,  Ranjitha Chacko, Jasmine Ruby Anand, Amala Rajan, Subhashini John, Vishalakshi Jeyaseelan, 2014, India | Qualitative Study to explore the end of life care perspective from the perspective of health care professionals as well as patients with advanced cancer in a tertiary health care setting in India | To highlight the high cost of inpatient care of decedents in comparison to that of survivors in case of out of pocket health care cost in India | Patients with advanced cancer and health care professionals | Patients put higher importance for emotional, social, physical and spiritual aspects of care as compared to professionals. 74 % of patients thought that discussion fear and anxiety is extremely important, 84.3% patients thought that quality of life conversation should happen at regular intervals with the health care team. 73% wanted to spend final hours at home, 95.7% of patients wanted others to pray for them, religion not withstanding. From Physicians aspect, 72.5% thought symptom management was extremely important and 62.5% felt need for emotional and social support.  This study shows that, there is need for medical professionals like doctors and nurses to take pro-active role in providing EOLC services and to ensure patient autonomy and facilitate good death. | Lack of Palliative Care Facilities: Palliative care facilities are not widely available in India, and there is no national palliative care policy, making it challenging to provide good end-of-life care.  Paternalistic Nature of Care: Despite the inclusion of patient independence in medical and nursing education, the paternalistic nature of care often undermines patient autonomy.  Advance Directives: The concept of advance directives is not well-developed in India. Most decisions are made by the patient's family due to the poorly developed social security and health insurance systems.  Resource Constraints: Lack of resources, illiteracy, poverty, and ignorance about available healthcare services make developing end-of-life care services difficult.  Emotional and Social Support: Patients place a high degree of importance on emotional, social, physical, and spiritual dimensions of care, but these needs are often not adequately addressed by healthcare professionals.  Financial Burden: Many patients feel they are a financial burden on their families, and there is a significant need for financial assistance to bear treatment expenses.  Communication Barriers: There is a lack of understanding of treatment options among patients, and they often feel unable to engage in intellectual conversations with healthcare providers.  Spiritual Needs: Patients have significant spiritual needs that are not always addressed by healthcare professionals, who may not consider spiritual care as part of their role.  Policy and Legal Framework: There is an urgent need for formulating an end-of-life care policy and legalizing advance directives in India.  Healthcare Professional Training: There is a need for healthcare professionals to be proactive in offering key supportive services to ensure patient autonomy and facilitate a good death. |
| 14 | End of Life Care Characteristics of the Elderly: An Assessment of Home based Palliative Services in Two Panchayats of Kerala,  Jayalakshmi R, Suhita Chopra Chaterjee, Debolina Chaterjee, 2016, India | Cross Sectional Survey | The study explores end of life characteristics of the elderly-socio-demographic status living patterns morbidity profile and functional status, it also looks at accessibility and utilization of palliative care services and respondents satisfaction with the services | Elderly patients from 2 panchayats-random sampling selected from palliative care service roster | Restricted functional mobility, prevalence of comorbid conditions which was higher in women compared to men, Financial insecurity, Lack of care, Functional dependence were key findings.50 % found praying as the only solution and hope. Access to palliative care services-field observations showed that 76 % were not satisfied with the services- due to attitude of care providers, irregular visits etc. Frequency of home visit was a major component of satisfaction.  Study poor quality of ageing and dying in Gram Panchayats of Kerala serviced by home based Palliative care, it also finds home based care in its present form may not be adequate to provide good quality EOLC. | Considerable Care deficit, especially home based financial constraint for these EOL patients which mean they even lack basic amenities like a proper home which adds to the distress level. Under such circumstances of considerable deprivation, the goal of home based palliative care to improve the quality of life may not be fulfilled. |
| 15 | Experiences in end of life care in Intensive Care Unit: A survey of resident Physicians  Zubair Umaer Mohamed, Fazil Mohammed, Charu Singh, Abish Sudhakar, 2016, India | Survey | The aim was to survey the attitude training and skills of intensive care residents in relation to EOLC | Intensive care residents with atleast more than a month experience with adults | 1. Experience in EOLC- 61.6% residents have cared for 10 or more dying patients, 40% have attended 10 or more EOL meeting with families, 48% residents have never communicated regarding organ donation to family of brain dead patients.  2. Communication- Residents were fairly comfortable in discussing prognosis and goals of care to critically ill patients.   3. Technical Skills- Most residents were comfortable with identifying a dying patient and managing their symptoms.  4. Training - Most residents felt they had a reasonable training in management of symptoms and communication during EOL. the significance was that the anaesthetic and surgical residents felt less trained.  5. Education- Most residents felt they could benefit with more training in EOLC symptom management and communication.  6. Attitude- Majority did not see withdrawal of support as euthanasia and did not have ethical, moral or religious regarding withdrawal of life support interventions.  The Study underlines the needs for learning requirements in EOLC skills to improve the quality of EOLC delivered in India. | Gaps seen were the need for enhanced training programs, better communication skills, and increased awareness of guidelines to improve the quality of End of Life care in intensive care units. |
| 16 | Attitudes of nurses towards care of dying patients in India  Rachelle J Lancaster, Cynthia Kautzmann, Jothi Clara J Micheal, Leena Chandrasekharan, Jayalakshmi Jambunathan, Tammy M Chapin, 2017, India | Pre and Post test questionnaire | To understand and describe nurses attitude when caring for dying patients in India and if participation in an educational activity which included 2 day scenario based, low fidelity simulations increase knowledge of end of life care | Nurses | Positive attitudes towards care of the dying, discomfort about communication regarding impending death with dying patient,uncertainity about addiction to Pain relieving medicines especially opioids especially in EOLC,Uncrtainity about assisting patients in dying phase and their families.  Religious, cultural and ethical factors are key points that influence the attitudes, knowledge of nursing working with and caring for EOLC patients, previous work or experience with EOLC may improve nurses' knowledge and attitude towards EOL care | Lack of Education/Knowledge: EOL and palliative care are not integral parts of professional undergraduate medical, nursing, or allied health professional curricula in India. Low baseline knowledge levels in several aspects of EOL care, particularly in resuscitation, use of morphine, pain management, and use of opioids.  Cultural and Religious Influences: Indian nurses' attitudes towards EOL care are influenced by cultural and religious beliefs, such as karma, suffering, reincarnation, and the sanctity of life, which can affect their support for practices like the withdrawal of life support.  Legal Ambiguities: There is confusion and fear around do-not-resuscitate (DNR) orders and passive euthanasia, which are illegal in India, leading to inconsistent EOL care practices.  Family-Centered Decision Making: EOL decision-making in India tends to be family-centered rather than individualized, which can complicate care.  Lack of Trust and High Costs: Families often take loved ones out of hospitals against medical advice due to a lack of trust, inadequate EOL care, or exorbitant costs. |
| 17 | Assessment of Private homes as spaces for the dying elderly  Tulika Bhattacharyya, Suhita Chopra Chatterjee, Dipannita Chand, Debolina Chattarjee, Jaydeep Sengupta, 2017, India | Qualitative Study | Assessment of end of life care of the elderly in private homes in Kolkata- India | Family caregivers | Themes that came out of this study were, a) Inadequacy of housing conditions as a space for dying patients b) Private homes may create an atmosphere for abuse of the sick and dying patients c) Care is provided mostly by females, especially daughters and daughter in law d) Lack of competence in caring for they dying patients at home e) Lack of palliative care support at home, where support was available it was partial due to financial constraints, long distance of travel and inadequate network in community. f) Challenges like lack of availability of medicines, lack of finances and knowledge & training in caring for sick and debilitated patients by the informal caregiver.  It was found that mostly females were responsible for the major workload in caring for the dying patients. Providing EOLC at homes is very challenging and the burden of care creates enormous stress on the informal caregivers. This study also showed that EOLC, when provided at home especially where families are dysfunctional can lead to abuse and conflict. | Architecturally Inadequate Housing: Many private homes are physically inadequate, with poor living conditions such as lack of space, ventilation, and sanitation, making it challenging to provide proper care.  Financial Constraints: There is a paucity of financial support, with most elderly relying on personal savings or family support. Health insurance coverage is minimal, adding to the financial burden.  Scarcity of Skilled Caregivers: There is a lack of skilled caregivers, with many families unable to afford or find competent help. This results in a significant care deficit, neglect ,abuse and exacerbated by the stress and burden on caregivers.  Peripheral Location in Public Health Framework: Private homes are not well-integrated into the public health system, leading to inadequate support and resources for EoL care.  Inadequate State Palliative Policy: There is a lack of a comprehensive palliative care policy, and stringent narcotic regulations hinder effective pain management.  Gendered Caregiving: The burden of caregiving falls predominantly on women, particularly daughters-in-law, leading to gendered stress and conflict.  Lack of Continuum of Care: There is a need for better integration of private homes with hospitals and other long-term care facilities to ensure a continuum of care.  Poor Access to Information and Support: Caregivers often lack access to necessary information, counselling, and supportive services, leading to confusion and inadequate care. Also support provided by NGOs is fragmented and inconsistent, with a need for better coordination and standardization of services. |
| 18 | Feasibility and acceptably of implementing the integrated Care Plan for the dying in the Indian Setting: Survey of Perspectives of Indian Palliative Care Providers  Navin Salins, Jeremy Johnson, Stanley Macaden, 2017, India | Survey | To understand the perceptions of Indian Palliative care providers regarding the feasibility and acceptability of implementing the international program for the best care of the dying in Indian setting | Professionals from 16 Palliative care centres who had attended the initial foundation course of the international collaborative for the best care of the dying person- purposive sample | Of the 30 participants, 21 completed the survey. It showed that, the few important aspects that came up were  a) Recognition of dying - symptoms and signs which recognized dying should be documented and discussed with patients and caregivers. b) A multidisciplinary team should be responsible for recognition of dying and decision making of EOLC planning c) Communication and information exchange regarding recognition of dying, all participants felt that dying person should be aware about the process and should be able to take part in the communication regarding the same. d) Spirituality- the dying person and their caregivers should be able to discuss their wishes, faith, values during this process.  e) Multidisciplinary team decision on medications, availability of medicines for symptom management in EOLC and anticipatory prescription of the same should be the norm. f) Multidisciplinary team decision on interventions - there should be review on use of blood investigations , antibiotics , Oxygen and vital sign monitoring at EOL. CPR should be reviewed and discussed at EOL. g) Clinically assisted nutrition and hydration- Review of assisted hydration and nutrition at EOL should be done and discussed with patient and family. h) Initial and Ongoing assessment- Recording and reviewing physical symptoms regularly, maintaining patients psychological and physical well being and maintaining the well being of the carer are the essential aspect of assessment. i) Communication regarding plan of care - explanation of the plan of care should be given to the patient and caregiver in addition a leaflet about the details (symptoms and ongoing changes before death) should be given to the caregiver. j) Care after death- verification of death should be done by health care professional, carers should be helped with the next procedures and legal documentation as part of the care plan.   The study showed that the majority of the items in the care plan with regard to EOLC were relevant to Indian setting. Palliative care providers in India need additional training for implementation of integrated care plan. Equipment's like syringe drivers has limited applicability in India.  "Development and implementation of care plan needs to be supported by education, quality improvement and research programme to ensure evidence based improvement in care of dying patients in India. | Limited Access: Only 0.4% of the population in India has access to palliative and end-of-life care.  Inappropriate Medical Treatment: Many people receive aggressive medical interventions at the end of life, leading to high treatment costs and lack of pain and symptom control.  Lack of Government Strategy: There is a poor government-led strategy towards national-level palliative care.  Training Deficiency: Health professionals lack adequate training in palliative care.  Shortage of Specialists: There is a shortage of specialist palliative care providers.  Public Funds: There is a limitation of public funds for palliative care.  Opioid Availability: There is a lack of availability of opioid analgesics.  Public Awareness: There is poor public awareness about palliative and end-of-life care.  Legal Frameworks: There are no clear legal frameworks or policies supporting clinicians in providing palliative and end-of-life care.  Multidisciplinary Team (MDT) Concept: The concept of MDT is rare and challenging to implement in the Indian setting.  Communication Challenges: It is often difficult to communicate the dying process to patients and their families due to cultural and logistical reasons.  Spiritual Care: Providing spiritual care is challenging due to diverse religious needs and lack of training.  Use of Equipment: Using equipment like syringe drivers for continuous infusion is challenging due to lack of familiarity and applicability in home care settings.  Life-Sustaining Treatment: There are ethical and legal challenges in limiting life-sustaining treatment and discussing cardiopulmonary resuscitation status. |
| 19 | Intensive care Nurses' attitude on palliative and end of life care  Swagara Tripathy, Pragyan K Rotary, Jagdish C Mishra, 2017, India | Self administered questionnaire | To investigate the knowledge, attitude and beliefs of intensive care nurses in eastern India towards end of life. | Nurses working in ICU | 1. Knowledge and attitude - 76.8% agreed that peaceful death in cases were further treatment is considered futile should be offered. 81.9% nurses agreed that they should be involved in EOL discussions with the family. 62.3% felt they should be the first to initiate EOL discussion with the family members, 53.6% agreed that continuing and feeding till a EOLC patient passes away is appropriate, 37 % felt relatives of EOLC patient should have full visitation rights. 2. 66% felt that inadequate counselling and 72% felt that religious and emotional sentiments of relatives and 44.2% agreed that disagreement between treating teams led to delay in EOLC implementation.  Experience in the ICU led to greater acceptance to withdrawal being similar to withholding and more involvement in witnessing EOLC discussions with families. There is a strong desire in critical care nurses to be involved in EOLC and palliative care in ICU's. | Younger nurses need further understanding of concepts of EOLC. There is a need of inclusion of relevant modules of EOLC and palliative care in nursing curriculum. Regular training is also warranted in ICU's to improve the scenario. Gaps identified were need for targeted education, ethical training, and improved communication strategies to enhance the quality of EOL care provided by ICU nurses. |
| 20 | End of life care and social security issues among geriatric people attending a tertiary care hospital of eastern India  Subraham Pany, Lipika Patnaik, E Venkata Rao, Sumitra Patnaik, Trilochan Sahu, 2018, India | Cross Sectional Study | To explore the desires of the elderly about making end of life care decisions and understand social security measures among the study population | Geriatric Patients(60+) attending outpatient department between Aug 2017 to Nov 2017 | 64% of participants expected to die at home in the presence of family members as compared to 32% in the hospital. Willingness of Organ donation after death was shown by 70% of the participants but only 6.5% registered for it. 94% of participants expected to be cremated and others buried as per religious practices. 7.24% had a legal will, 48% of the participants did not have any form of health or life insurance.  Need of Home based palliative care for the elderly dying patients. It was seen in this study that there was a denial to talk about the death because ideology of death should not be discussed.  It also showed that there will be financial burden for a near and dear one's of the dying patients. | Lack of social support for the employed and unemployed poor. Lack of information about insurance (Health & Life) Lack of information about legal documentation (Will) Low rate of registration for organ donation |
| 21 | "End-of-Life Care is more than Wound Care"- Healthcare providers perception of psychological and interpersonal needs of patients with terminal cancer  Nisal Pinto, Poornima Bhola, Prabha S Chandra, 2019, India | Qualitative Study | Exploring health care providers perceptions about psychosocial interpersonal and unfinished business of the terminally ill cancer patients during the end of life care | Health care workers with minimum 6 months work in palliative care centre | The themes derived -  a) Psychological needs and concerns - experience and expressions of negative emotions, mental health concerns, confronting mortality b) Interpersonal connections - Support and closed connections of family, a disconnect from family relationships, new connections at the hospice c) Unfinished Business- Types of unfinished business, addressing unfinished business d) Professional Caregiver perspectives and experiences - need for expanded EOLC training, experiences of emotional labour.  The study showed that patients have different interpersonal needs, they require support and deeper connections with their families at the same time as they try to avoid or detach from family bonds.  " Need for belongingness and sexual intimacy with the partner emerged as an unmet need in this study".  This study also showed the reluctance in patients in discussing body image issues, fears of rejection from partner, minimal communication about sexual needs. Health care providers in this study showed a sensitivity to the experience of negative emotions and mental health vulnerabilities of their patients as they approached EOL. They expressed the challenges that health care providers dealt with when patients went through a gamut of negative emotions like anger and aggressive behaviours.  Using communication skills to understand the scoping mechanism can help in reaching out to angry dying patients more empathetically and effectively. | Inadequate Training: Many health-care providers lack formal training in end-of-life care, particularly in dealing with patients' emotions, breaking bad news, and understanding religious and subcultural beliefs and rituals.  Psychological Needs: Psychological needs such as anxiety, depression, and suicidal thoughts are often underrecognized and inadequately addressed. There is a need for rapid assessment tools and ongoing training for health-care providers to manage these concerns effectively. Interpersonal Needs: Patients have diverse interpersonal needs, including the need for closer connections with family and sexual intimacy, which are often unmet due to barriers in communication and hospice settings.  Unfinished Business: Patients often have unresolved issues or "unfinished business" related to life choices, roles, responsibilities, and reconciliation with family members.  Emotional Labor: Health-care providers experience significant emotional labour, leading to compassion fatigue and burnout. There is a need for systemic support and self-care practices for these providers.  Cultural Sensitivity: There is a need for culturally sensitive care that considers the varied religious and subcultural belief systems of patients, especially in a diverse country like India.  Communication: Effective communication with patients and their families about the dying process and patients' wishes is often lacking. |
| 22 | End of Life decisions: A retrospective study in a tertiary care teaching hospital in India  Cijoy K Kuriakose, Vignesh Kumar Chandiraseharan, Ajoy Oommen John, Deepti Bal, Visalakshi Jeyaseelan, Thambu David Sundarsanam, 2019, India | Retrospective Observational Study | Assess factors associated with EOLC decisions and raise awareness about EOLC | Patients who died in hospital in department of Medicine between Oct 2014 and Sept 2015 | 66.4%patients had withdrawing or withholding of life support, out of which 76% was withholding of life support and 23% was withdrawal of life support. 50% of deaths were due to sepsis, 29.5% due to type-1 respiratory failure, 64.23% died within less than 1 week of admission. Documentation of disease process, prognosis and mention of imminent death were factors associated with decision of WWLS(withholding or withdrawal of life support).  A significant proportion of deaths occurs in hospitals and especially in ICU's. Lack of awareness of EOLC may lead to aggressive interventions at EOL. Decision to withdraw or withhold is usually taken by patient and their families by consulting the physicians based on the financial and family conditions. Long stay in hospital/ICU, educational/professional status. Physician based decision making on WWS is dependant on prediction of poor outcome, neurology, neuro-surgical issues and the assumption that the patient does not want life support. | The study highlighted the use of futile medical interventions at EOL  Thel ack of use of APACHE-II scale for critically ill patients results in lack of objective data regarding their prognostication. Lack of Clarity and Data: on the factors influencing EOL decisions in India.  Documentation: documentation of poor prognosis was the only significant factor associated with the decision to withhold or withdraw life support. This indicates a gap in the comprehensive documentation of other influencing factors. |
| 23 | End of Life care practices in rural south India: Socio cultural determinants  Sudha Ramalingam, Subhashini Ganesan, 2019, India | Qualitative Study | To explore EOLC practices with regard to Social, Cultural and Religious dimensions associated with those practices in Rural Tamil Nadu | Permanent residents of the rural area, aged above 40 Yrs. with experience in taking care of terminally ill or sick elderly in their house | The study showed existence of practises like "THALAIKOOTHAL" in rural area for terminally ill patients. Some of these rituals hasten death by hypothermia, renal failure or aspiration pneumonia. These practices are influenced by socio-economical, cultural, moral and spiritual factors (lack of financial-social support, social restrictions due to sick person in the family, customs regularly practiced and participants perceptions towards moral duties towards parents and elders, spiritual perceptions that dying person can attain god through these practices).  The study highlights cultural beliefs, dependence on customs, moral and spiritual perceptions behind practices like " THALAIKOOTHAL" which lead to death of a terminally ill patient. Some of these EOL practices are meant to minimize suffering and a peaceful death.  This study also highlighted that closed communities encourage these practices and the moral obligation of the family to ensure that a terminally ill patient dies a peaceful and dignified death. The discussion also showed the communities perception that " Only good death can help people reach GOD". | Need for community based palliative care support in the rural areas and effective palliative care approach can reduce such practices in EOL. Also, education and empowerment of the communities towards EOLC provision.  This study shows that in the guise of dignified death, the practice of "THALAIKOOTHAL" is an act of euthanasia and the suffering that the elderly terminally ill patients undergo cannot be disregarded |
| 24 | Knowledge and Awareness of End of Life Care among Doctors Working in Intensive Care Units at a Termitary care centre: A questionnaire based study  Krithika Agrawal, Rakesh Garg, Sushma Bhatnagar, 2019, India | Questionnaire based study- Cross sectional | To evaluate the knowledge and awareness of doctors working in critical care units towards EOLC | Doctors working in critical care units with more than 03 months of experience | 1. Experience with EOLC situation- 80% respondents did not have experience of counselling family member regarding organ donation.  2. General awareness and knowledge of EOLC- 81.7% had heard of EOLC but most lagged knowledge of EOLC, only 11.4 % had taken training related to EOLC, 61.9 % felt that patients and families have the right to choose to discontinue life support interventions. More than 50% felt that providing EOLC required emotional detachment. Those with more experience were more aware of EOLC and awareness were more in doctors from anaesthesia, Critical Care, Pulmonary Medicine as compared to Neurology and Internal Medicine. It was statistically significant that awareness of good death paralleled with awareness of EOLC with designation.  EOLC needs to be an integral part of critical care management and curriculum should include EOLC training as well. | Lack of awareness about Indian guidelines about EOLC highlights the need for better education and dissemination of EOL training. Lack of specific EOLC training in medical curriculum. Gaps highlighted are the need for improved training, better dissemination of guidelines, and inclusion of EOLC education in medical curricula to enhance the competence and comfort of doctors in managing end-of-life care. |
| 25 | Family Caregivers Experiences with Dying and Bereavement of Individuals with Motor Neuron Disease in India,  Manjusha G. Warrier, Priya Treesa Thomas, Arun Sadasivan, Bhuvaneshwari, Balasubramaniam, Seena Vengalil, Saraswati Nashi, Veeramani Preethish-Kumar, Kiran Polavarapu, Niranjan Prakash Mahajan, Pradeep Chandra Reddy Chevula & Atchayaram Nalini, 2019, India | Qualitative study | The primary aims of the study were to explore  (a) the caregivers 'experiences of the end-of-life stage, and (b) the sources of support for individuals and their caregivers with MND at the end-of-life stage. | Bereaved caregivers of individuals with MND from national tertiary referral care center | Themes derived were transition from person to patient, 1. The caregivers remembers the patient as a person before and after onset of disease. 2. Support during advance stage- lack of readiness or willingness of patient to avail services restricted support. Patients choices were given importance in most cases and few did not want life prolonging treatment. 3. Death- Caregivers despite knowing the course of the illness were not prepared for death.  4. Impact on Caregivers- Significant impact and carer fatigue was noted especially due to loving hours of care leading the caregivers to be emotionally, physically and financially exhausted.  The study highlights the importance of social worker as a care manager for patient and family, strong community orientation, networking are required in such care taking. | Lack of Awareness and Readiness: There is minimal awareness among the public and primary health practitioners about MND and the need for palliative care, making management at home and in the community challenging.  Limited Access to Palliative Care: Only 1% of those who need palliative care receive it. There is a lack of uniform palliative care policy and limited institutionalized support or hospice care . Financial Burden: Healthcare costs are a significant burden, with most expenses met out of pocket due to inadequate health insurance coverage. This financial strain can lead to impoverishment.   Support Systems: The support systems available during advanced stages of illness are inconsistent, and the lack of a care manager to coordinate care is evident.  Cultural and Religious Barriers: Cultural and religious beliefs can act as barriers to discussing and planning for end-of-life care, leading to inadequate preparation and support. |
| 26 | Quality of Death: The unspoken experiences of patients with advanced cancers in India- An exploratory qualitative study  Revathy Sudhakar;Surendran Veeraiah;Prasanth Ganesan, 2020, India | Qualitative study | To explore the experiences and perceptions about quality of death of caregivers of patient diagnosed with advanced cancer | Caregivers of patients with advanced cancers who died at home or during hospitalization | Of the 108 caregivers- 79 of the patients died at home and 29 at hospital. Themes that emerged from the study about QOD were- 1. Bodily discomfort (unbearable pain/breathing difficulty lack of intake). 2.Psycho-social experiences- both poor and good experience depending on the variables. 3.Resilience- able to accept the disease.  4. Existential distress- fear, worry suicidal ideation, hopelessness, crying spells were seen in patients of half the study population. 5. Prognostic Awareness- caregivers felt that the patients were aware of their deteriorating state. 6.Aware- patient aware of prognoses were more stable psychologically and able to cope. 7.Carer's coping- perceived strain and negative emotions, contentment for being able to take care of the patient till end.  Study underscores the fact that patients and their caregivers in EOL require more psycho-social support and improved symptom management | Physical Symptom Management: Many patients experience severe physical discomfort, including pain, difficulty in breathing, eating, and mobilizing, which are not adequately managed.  Psychosocial Support: There is a lack of sufficient psychosocial support for both patients and caregivers, leading to significant psychological distress.  Prognostic Awareness: Many patients are unaware of their prognosis, which can lead to increased emotional distress and poor psychological health.  Cultural Stigma: The stigma associated with discussing death and EOL care in India prevents open conversations and planning, which can negatively impact the quality of death (QOD).  Caregiver Strain: Caregivers experience emotional and physical strain, feelings of guilt, frustration, and exhaustion, indicating a need for better support systems for caregivers.  Palliative Care Availability: Palliative and hospice care services are limited and concentrated in certain regions, leading to disparities in the quality of EOL care.  Communication and Decision-Making: There is a need for improved communication between healthcare providers, patients, and caregivers to discuss prognosis, treatment preferences, and EOL care options. |
| 27 | Barriers, Facilitators and recommended strategies for implementing a home based palliative care intervention in Kolkata  Suparna Qanungo, Alejandra Calvo-Schimmel, Shannon McGue, Pooja Singh, Rakesh Roy, Gautham Bhattacharjee, Nibedita Panda, Gaurav Kumar, Rekha Chowdhury, Kathleen B Cartmell, 2021, India | Qualitative Study | To identify barriers, facilitators and recommended strategies to develop a home based palliative care intervention for poor and people with lack of medical access in rural Kolkata- India | Doctors, Cancer centre administrators, Health care workers and patient and patient care givers | Themes identified, a) Overview of context of palliative care delivery- Underdeveloped palliative care mostly available only in cancer centres, home care available in limited areas near cancer centres, poor patients living in far and remote areas do not have access to palliative care, lack of government resources. b) Barriers to delivery of palliative care- Patients do not access palliative care until EOL, many patients not aware about terminal stage of the cancer, financial constraints due to low income or lack of health insurance, exorbitant cost of medications and long distance of travel to get access to these medicines. Under and lack of appropriate use of medicines especially when patients when patients stop use of medicines when symptoms improve, barriers towards morphine usage due to limited availability, misconceptions by both patients family and medical fraternity, transportation long distances and lack of available transport services and funds may lead to difficulty in availing adequate palliative care. Problems associated with family dynamics like concerned about financial and social burden on family, lack of family member for assistance, unsupportive or dysfunctional families, Resistance to health care workers visiting home due to fear of isolation and ostracism by community. Resistance from local doctors towards delivering palliative care. Local doctors fear competition or RMPs as unsuitable to provide palliative care to patients at home. c) Facilitors for delivery of palliative care - existing palliative care at cancer centre medications and services are provided at free of cost, low cost and availability of morphine, sources of support for patient-family is the most of important source of practical and emotional support to patient. Limited support of private organizations and government. d) Recommended Strategies for delivery of Palliative Care - Different models of palliative care, creating distribution points for morphine access and training of RMPs (Rural Medical Practitioners) to deliver palliative care.   This study highlighted that health system barriers especially lack of available medicines like morphine, lack of access to palliative care and socio-cultural barriers. India despite being one the major producers of morphine in the world, the use and procurement of morphine is difficult for palliative care patients. Late referrals, lack of awareness about the disease and prognosis, family and cultural dynamics, absence of reliable health system, misconceptions and beliefs about cancer as major factors leading to poor PC in most areas. | Limited Access to Palliative Care: Only a small fraction of the population in need of palliative care receives it, with significant barriers in rural areas.  Late Diagnosis and Referral: Patients often seek care at advanced stages of illness, and oncologists tend to refer patients to palliative care late in the disease course.  Financial Barriers: High treatment and medication costs, lack of health insurance, and transportation expenses pose significant challenges.  4Medication Access: There are issues with the affordability and availability of medications, particularly in rural areas.  Cultural and Social Barriers: Stigma around cancer and morphine use, reluctance to discuss terminal illness, and family dynamics can hinder effective palliative care delivery.  Healthcare System Barriers: There is an underuse of certain medications like antidepressants, insufficient primary care and oncology specialists, and resistance from local doctors towards using rural medical practitioners (RMPs) to deliver palliative care.  infrastructure and Resource Limitations: There is a lack of government resources and support for palliative care, and existing services are often underdeveloped. |
| 28 | Clinical and Socio-demographic profile of hospice admissions in New Delhi, India  Astha Koolwal Kapoor, Sushma Bhatnagar, Rajni Mutneja, 2021, India | Retrospective Study | Assess the clinical and demographic profile of patients admitted to a hospice in New Delhi between 2016 and 2017 | Hospice patients | 60% of the patients were females, majority of the admissions were from Delhi The median hospice stay was approximately 42 days with longer stays for female patients. Death information was available for 67% of patients, majority of them had occurred in the hospice. Only 77% of the patients were aware about their prognosis Most common cancers were head & neck followed by GI and breast cancer 66% of them received opioids 39% of the caregivers were children of the patients and 32% were spouses.  The study showed that age of admission to hospice was younger was compared to western countries, patients with poor socio-economic status have more access to hospice due to lack of proper housing and also the hospices provide free care to these patients. | Influence of socio-economic conditions status on death is different from the west, the helplessness and lack of training of caregivers in dealing with difficult symptoms at EOL. Gaps highlighted are the need for more comprehensive and detailed research to improve EOL care and inform policy decisions in India |
| 29 | Awareness and attitudes of primary care givers towards end of life care in advanced cancer patients: A cross sectional study  Sourav Burman, Rakesh Garg, Sushma Bhatnagar, Seema Mishra, Vinod Kumar, Sachidinand Jee Bharathi, Nishkarsh Gupta, 2021, India | Prospective Cross Sectional Observational Study | To Assess awareness about an EOLC in caregivers of advanced cancer patients | Primary Caregivers of patients who were undergoing palliative treatment and who were denied cancer curative therapy | 26% of the caregivers were aware of the term palliative care, female caregivers were more knowledgeable about EOLC and 68% of them were willing to initiate EOLC. 47% of the rural population were more aware and willing to adopt palliative care and receptive towards stopping aggressive therapies.  This study showed that most people were willing to learn about EOLC and believed that information should be available and shared more frequently. Female caregivers were more prone towards initiating EOLC.  This study also shows that most caregivers wanted to avoid resuscitation and ventilation for terminally ill patients in ICU.  The study also found poor awareness of palliative care and EOLC in the general population The rural population was more amenable towards initiating EOLC in terminally ill patients and foregoing aggressive therapies and futile interventions mostly due to long duration of suffering, increased financial constraints and lack of human resources. This study also showed that 98% of caregivers felt financial constraint as an issue and most of the caregivers wanted to opt for institutionalized care due to unwillingness to take these patients home. | Low Awareness of EOLC and gender disparity: Only 26% of caregivers were aware of the term palliative care, indicating a significant lack of knowledge about EOLC among caregivers. Female caregivers were more knowledgeable about EOLC compared to males, with 68% of females willing to initiate EOLC.  Rural vs. Urban: The rural population was more willing to adopt palliative care (47%) and more receptive to discontinuing aggressive therapy compared to the urban population.  Educational Influence: higher education levels had better awareness of EOLC, but there was still a lack of practical application among the educated urban population.  Financial Burden: Financial issues were a significant concern Cultural and Social Taboos: There are cultural and social taboos around discussing death, which hinder the acceptance and implementation of EOLC.  Lack of Information Sources: The primary source of information about palliative care was from friends or relatives who were cancer survivors, indicating a lack of formal education and awareness programs.  Preference for Institutional Care: Despite the desire for comfortable EOLC, many caregivers preferred institutional care over home care, highlighting a gap in the availability and acceptance of home-based palliative care services.  Misconceptions about Palliative Care: There is a prevalent myth that EOLC hastens death, which affects the willingness to adopt palliative care practices.  Need for Better Communication: Effective communication and involvement of caregivers in decision-making are crucial for better acceptance of EOLC. |
| 30 | An Observational study on the effects of delayed initiation of EOLC in terminally ill young adults in the intensive care units  Anirban Honn Choudhuri, Sakshi Duggal, bhuvana Ahuja, Rajeev Uppal, 2021, India | Retrospective Study | To measure, identify and compare the prevalence and risk factors for the delayed initiation of EOL in terminally ill young adults | Terminally ill young adults between the age group of 20-40 Yrs. admitted between June 2014 and Nov 2018 in a 07 bed ICU which were treatment futile | 39% of the participants were in normal group whereas 61% were in late group. The normal group had lesser drug consumption and ICU resource usage that was 14.7%. The social and family support of relatives of the n-group was higher as compared to late-group.  Study found delayed initiation of EOL in terminally ill young adults, some of the reasons were of non-acceptance of EOL in the hope of miraculous recovery, lack of understanding of basic principles of EOL, validation of EOL from family members to support the decision and fear and apprehension of continuing care at home in the event of discharge. | Delayed Initiation: A significant proportion of patients (61.1%) experienced delayed initiation of EOL care, starting more than 24 hours after the recognition of treatment futility.  Nonacceptance and Belief in Miraculous Recovery: Many patients and their families did not accept EOL care due to a belief in miraculous recovery and a denial phenomenon.  Lack of Family and Social Support: Adequate family and social support were lacking, which contributed to the delay in initiating EOL care.  Educational and Societal Factors: Lower levels of education and societal background influenced the delay, as those with higher education and better social support were more likely to consent to EOL care earlier.  Psychological Distress: The study highlighted the need for better management of psychological distress, including anxiety, depression, and existential concerns, which were not adequately addressed.  Resource Utilization: Delayed initiation led to higher drug consumption and ICU resource usage, which could have been reduced with earlier EOL care. Counselling Quality: There was a gap in the quality of counselling provided, as initial counselling by senior physicians followed by resident doctors and nurses might have led to inconsistencies. |
| 31 | Truth-Telling to Terminal Stage Cancer Patients in India: A Study of the General Denial to Disclosure,  Souvik Mondal, 2021, India | Mixed Methods Study | To understand the nature and practice of truth telling to adult patients suffering from terminal stage of cancer | Adult cancer patients above 18 years in age, caregivers and physicians(May 2017 to December 2019) | Of the 118 patients interviewed 85.60% preferred full disclosure, 8.4 % did not want to know, but would like the family to Know, whereas 5.9% did not wish either family or themselves to be aware about the disease. The younger patients between 18-35 years wanted full disclosure. No significant difference between genders were found where disease disclosure was considered. However when family members of these 118 patient were interviewed almost 77.12% expressed reluctance towards full disclosure. All 25 physicians were ethically agreeable on full disclosure to patient but 96.6% of the 25 physicians disclosed the diagnoses to the family first, considering the importance of family in Indian setting.  This study found that 22.3% of patients are fully aware about their terminal stage disease | The need for full disclosure to patients about their disease status especially since it helps in decision making. The collusion between the family members is mostly due to the fact that they would like to protect the patient from further distress even though they feel that telling them is the right thing to do. The physicians in general respect the family centric culture and the families authority to make those decision though they ethically feel that the patient should be told about their disease status |
| 32 | Cross Country Comparison of Expert Assessments of the Quality of Death and Dying in 2021.  Eric A. Finkelstein,Afsan Bhadelia, Cynthia Goh, Drishti Baid,Ratna Singh, Sushma Bhatnagar, and Stephen R. Connor, 2022, Cross Country | Survey on 13 key indicators of EOLC | To quantify delivery of EOLC in different countries. | 181 experts from 81 countries | India stood 59th position in quality of death ranking.  Policy makers need to take measures to improve EOLC delivery like increasing access to opioids and other essential medications to reduce health related suffering, Services to avoid medical bankruptcy, Education and Training programmes to increase capacity to deliver high quality EOLC, It also highlights the factors like Public Education Programmes, Promotion of Compassionate Communities and higher investments in research which will indirectly improve EOLC | Access to Palliative Care: Many low and middle-income countries lack basic access to palliative care, leading to patients dying in pain and distress.  Quality of Care: Even in high-income countries, EOL care often fails to meet important patient and caregiver needs, such as pain management, emotional support, and dying at the place of choice.  Disparities: There are significant disparities in the quality of EOL care between high-income countries and others, with low-income countries generally performing worse.  Integration into Health Systems: Limited integration of palliative care into broader health systems, resulting in fragmented and uncoordinated care.  Policy and Investment: Lack of national strategies, policies, and investments in palliative care, leading to inadequate resources and infrastructure.  Education and Training: Insufficient training and education for healthcare providers in palliative care, resulting in a shortage of skilled professionals.  Public Awareness: Low public awareness and understanding of palliative care options, leading to underutilization of available services.  Financial Barriers: High costs of EOL care can be a barrier, preventing patients from accessing necessary services.  Cultural Factors: Lack of recognition and accommodation of cultural factors associated with death and dying.  Communication: Poor communication between healthcare providers, patients, and families about EOL care options and decisions. |
| 33 | Stakeholder engagement as a strategy to enhance palliative car involvement in intensive care units: A theory of change approach  Seema Rajesh Rao, MSc, MBBS,Naveen Salins, PhD, MD, FRCP, Bader Nael Remawi, MSc, et. All | Theory of Change- Palliative care integration in ICU's in India | It explores a strategy to improve palliative care engagement in ICU through a stakeholder participatory approach | Stakeholders representing anaesthesia, emergency medicine, critical care, internal medicine and palliative care | The study identified 6 outcomes and the anticipated impact of greater collaboration between intensive care and palliative care clinicians in ICU's to provide better end of life care and good death. Palliative care engagement in ICU's improved patient centred communication, care plan discussions and documentation of advanced directives including DNAR preferences. Post strategies were identified as part of theory of change map 1. Developing an educational program for health care professionals on palliative care in ICU as per EPEC template. 2. Develop patient and family information guide and conduct public awareness program on palliative care and critical illness 3. Weekly webinar on palliative care in ICU with both palliative care and intensive care inputs. 4. Develop hospital end of life care quality with ICU focus. | 1. Limited Literature: There is limited literature on palliative care engagement in ICUs, especially in lower-middle-income countries like India.  2. High Symptom Burden and Costs: Adult patients in the terminal phase in ICUs experience high symptom burden, increased costs, and diminished quality of dying.  3. Lack of Awareness and Policy: There is a lack of awareness and policy regarding palliative care in ICUs, which hinders its integration.  4. Insufficient Providers and Administrative Support: There is a shortage of palliative care providers and inadequate administrative support and planning. 5. Sociocultural, Religious, and Economic Barriers: These barriers contribute to the patchy implementation of end-of-life care in Indian ICUs.  6. Legal Conundrum: The legal issues associated with withholding and withdrawing life-sustaining interventions complicate the implementation of palliative care.  7. Discharge Against Medical Advice (DAMA/LAMA): High personal costs lead to frequent discharges against medical advice due to a lack of funds to sustain ICU treatment.  8. Lack of Symptom Assessment: Pain, delirium, and sedation are assessed in less than half of the patients in ICUs. |
| 34 | Impact of End-of-Life Nursing Education Consortium on Palliative Care Knowledge and Attitudes Towards Care of Dying of Nurses in India: A Quasi-Experimental Pre-post Study  Mayank Gupta, MD Gegal Pruthi, MD, DM, Priyanka Gupta, MD, Karamjot Singh, MDataSc,Jyoti Kanwat, MD and Avinash Tiwari, MD | Experimental Pre-post study on ELNEC training | Access the impact of ELNEC training on the knowledge and attitude of nurses in India towards palliative care and care of the dying | 108 registered nurses | Most nurses had poor baseline palliative care knowledge. Still, many exhibited better information in pain and symptom management due to increasing emphasis on pain assessment as the fifth vital sign.  There was poor knowledge about spiritual and psychosocial care. Most nurses had favorable attitudes towards training in palliative and end-of-life care. The post-test study findings showed improved knowledge and attitude in nurses, proving that short-term educational courses like ELNEC are a good alternative to prepare nurses in EOLC in palliative care. ELNEC program effectively enhanced nurses' attitudes by making them more comfortable, supportive, and positive toward the care of dying patients and their families | Absence of Palliative Care Training: Palliative care training is conspicuously absent in Indian nursing curricula, a significant obstacle to delivering quality end-of-life care.  Insufficient Knowledge: The baseline knowledge of practicing nurses in palliative care was poor, as evidenced by low scores on the Palliative Care Quiz for Nursing (PCQN).  Inadequate Focus on Psychosocial and Spiritual Care: The study highlighted a need for increased focus on palliative care's psychological and spiritual aspects, as these areas had the fewest correct answers in the PCQN.  Need for Structured Educational Programs: The study emphasized the necessity of starting structured educational programs to improve the knowledge and attitudes of practicing nurses in palliative care and end-of-life care. |
| 35 | Exploring Perception of Terminally Ill Cancer Patients about the Quality of Life in Hospice based and Home based Palliative Care: A Mixed Method Study  Dhriti Patel , Parimalkumar Patel,, Monal Ramani, Khushbu Makadia | Mixed Methods Study | Evaluate the perception and performance of terminally ill cancer patients for the quality of palliative care in different settings and measure quality of life at end-of-life | 68 terminally ill patients | Most patients preferred hospice-based care over home care based mainly on the clinical skills, expertise, and round-the-clock care available in a hospice.  Home-based care, though welcomed, was lacking due to irregular visits and fear of escalating symptoms without the availability of immediate support. | Need for quality palliative care services that are home-based. Patients preferred to stay in inpatient/hospice setup out of fear of being neglected or not receiving adequate emotional and psychosocial support at home, especially where symptom management is concerned |
| 36 | A Nationwide Survey on the Practice of End-of-life Care Issues in Critical Care Units in India  Indu Kapoor, Hemanshu Prabhakar, Charu Mahajan, Kapil Gangadhar Zirpe, Swagata Tripathy, Jaya Wanchoo, Gaurav Kakkar, Harsh Sapra, Nidhi Gupta, Vasudha Singhal, Arvind Chaturvedi | Survey | Assess the practices of end-of-life care in critical care units in India | 91 clinicians | The study showed that years of experience and practice settings of clinicians had an effect on various treatment strategies and approaches to prognostication in terminally ill patients.  Clinicians with years of experience were more prone to discuss care/management plans with relatives, address symptoms, regularly access patients, and also provide bereavement support.  Clinicians working in urban setups were more frequent in providing palliative sedation, recognizing end-of-life, and imposing more restrictions on visitors in ICU, whereas clinicians in rural settings reported higher overall satisfaction with pain and symptom management, and patients were more informed and emotionally supported.   This survey also showed that regardless of the practice area the care plan was almost always discussed with relatives and very few with patients themselves.  108 practice centers were identified that provided end-of-life care; most were NGOs, Private hospitals, and Hospices. It was observed that withholding or withdrawal of care in terminally ill patients was highest among nonteaching private institutes than Govt centers. | Experience and Training: Less experienced clinicians provide lower quality EOL care compared to their more experienced counterparts, indicating a need for better training and mentorship.  Communication: Inconsistent communication with patients and their relatives about the terminal status, with many clinicians not always discussing care plans directly with patients.  Spiritual and Psychological Support: Inadequate provision of spiritual and psychological support to terminally ill patients.  Palliative Sedation: Variability in the use of palliative sedation, with some clinicians not providing it consistently.  Withholding and Withdrawal of Care: Inconsistent practices regarding withholding or withdrawing care, often due to legal issues, lack of hospital policies, or lack of awareness.  Dedicated EOL Teams: Lack of dedicated teams in ICUs to handle EOL issues.  Social Care Workers: Limited involvement of social care workers in handling grief among terminally ill patients and their families.  Patient Wishes and Will: Clinicians often do not ask patients about their last wishes or discuss their will with relatives. Resource Constraints: Challenges such as space, staff, and lack of hospital policies affecting the provision of EOL care. |
